# Markers of Prenatal Androgen Exposure Correlate With Online Sexual Compulsivity and Erectile Function in Young Men

**DOI:** 10.3389/fpsyt.2021.517411

**Published:** 2021-04-06

**Authors:** Verena N. Buchholz, Christiane Mühle, Cohort Study on Substance Use Risk Factors, Johannes Kornhuber, Bernd Lenz

**Affiliations:** ^1^Department of Psychiatry and Psychotherapy, Friedrich-Alexander University Erlangen-Nürnberg (FAU), Erlangen, Germany; ^2^Department of Addictive Behavior and Addiction Medicine, Central Institute of Mental Health (CIMH), Medical Faculty Mannheim, Heidelberg University, Mannheim, Germany

**Keywords:** prenatal androgen load, 2D:4D, digit ratio, spermarche, pornography addiction, premature ejaculation, erectile function, behavioral addiction

## Abstract

Pornography addiction and sexual dysfunction are increasingly prevalent in young men. Previous studies suggest that prenatal androgen exposure plays a role in addiction and sexual functionality. Here, we tested whether lower second-to-fourth finger length ratio (2D:4D) and later age at spermarche, both putative indicators of higher androgen levels *in utero*, correlate with online sexual compulsivity (OSC scale of ISST), erectile function (IIEF-5), and ejaculatory control (PEPA) in 4,370 young men (age IQR: 25–26 years) of the Cohort Study on Substance Use Risk Factors. Statistical analyses revealed that lower 2D:4D correlated with higher scores on the OSC scale. Moreover, higher age at spermarche correlated with higher OSC scores and decreased erectile function. Interestingly, OSC severity, but not the frequency of pornography use, correlated negatively with erectile function and ejaculatory control. This is the first study to associate two independent proxies of prenatal testosterone level with OSC. These findings provide novel insight into intrauterine predisposition of sexual behavior and related sexual function in adulthood.

## Introduction

A growing body of research supports that pornography addiction causes a great burden particularly to young males (1, 2). However, due to different conceptional categorizations and self-report bias, prevalence estimates are imprecise. Today, little is known about the biological mechanisms underlying pornography addiction.

Excessive pornography use is considered to promote sexual dysfunctions [for review, see (3)]. Erectile dysfunction affects primarily men above 40 years of age with previously reported prevalence rates of 1–10% in younger men and 50–100% in males older than 70 years (4). However, psychogenic erectile dysfunction in men under 40 years has risen sharply in the last decade up to rates as high as 14–28% in Europeans aged 18–40 years (5–7). The drastic worldwide increase of pornography use as sexual stimulation has been discussed to induce erectile dysfunction *via* alterations in the brain's motivational system (mesolimbic dopamine pathway) (3). Erections depend on dopaminergic neurons in the ventral tegmental area (VTA) and dopamine receptors in the nucleus accumbens (NAc) (3, 8, 9). This reward system is highly activated during pornography viewing with alterations in brain connectivity to the prefrontal cortex observed in subjects with pornography addiction compared with controls (10). Also, other addiction-related phenomena, like increased cue sensitivity, are observed in the brain responses of individuals addicted to pornography (11). Pornography has a high potential for addiction, considering its accessibility, affordability, and anonymity (2). Addiction to it can lead to a cascade of problems, ranging from erectile dysfunction to low sexual desire in partnered sex and relationship problems (3). Although clinical reports often suggest function improvement after abstinence from pornography, direct evidence of a causal effect is lacking (3), as is a scientific understanding of compulsive pornography use and its associated dysfunctions. For organic erectile dysfunction, in contrast, cardiovascular risk factors represent strong predictors (4).

Ejaculatory control also seems to be affected by excessive pornography consumption in hypersexual patients, resulting in reports of ejaculation difficulties in 33% of the patients (12). Premature ejaculation occurs frequently in adolescent males, especially during their first sexual encounters (13) and decreases over time as experience confers increased control. The criteria for premature ejaculatory control, according to the International Society of Sexual Medicine, are fulfilled by only 4–5% of the worldwide population. Furthermore, the perception of premature ejaculatory control is influenced by social conditioning through pornography consumption (14).

Males are more prone to pornography addiction than females (15). An Australian study found a self-reported prevalence rate of 4% in 9,963 men and only 1% in 10,131 women. This sex-based difference is also present in other non-substance-related and substance-related addictions, such as gambling (16), internet gaming (17, 18), and alcohol dependence (19). In general, sex differences originate from the sexual imbalance in X and Y chromosomes which determine gonadal development and later secretion of androgens and estrogens. During sensitive windows (e.g., prenatal, perinatal, and pubertal), these sex hormones lead to permanent organizational effects on brain and behavior which are discriminated from direct and reversible activational effects (20). Thus, studies have investigated the role of prenatal androgen exposure underlying addictive behavior. Indeed, initial associational evidence has suggested that video gaming addiction (21) and alcohol dependency are (22, 23) both related to prenatal androgen exposure. Together with genetic evidence linking sex hormone signaling to dependency (24–28), this suggests that androgen activity is involved in the pathology of addiction. Furthermore, a rodent study provides direct evidence that prenatal androgen receptor modulation affects alcohol consumption during adulthood (29). Human studies based on indirect markers of prenatal androgen exposure support its prenatal role in the development and maintenance of addictive behaviors during adulthood. Direct investigations of this issue in humans are hardly feasible due to ethical concerns and the long interval between the prenatal period and adulthood.

Research based on rodent experiments and human associational studies has identified markers of prenatal androgen level, such as the second-to-fourth finger length ratio (2D:4D) [(30, 31); but see also: (32, 33)] and age at first ejaculation (spermarche) (34, 35). Human maternal plasma testosterone levels are negatively correlated with newborns' digit ratio in both sexes (36), and amniotic fluid testosterone levels are negatively associated with 2-year-olds' 2D:4D (37). A recent meta-analysis found lower 2D:4D (indicative of increased prenatal androgen exposure) in males with substance-related and non-substance-related addictive behaviors (Hedge's *g* = −0.427) but not for females (Hedge's *g* = −0.260). This effect was stronger in the sub-analysis comparing dependent with non-dependent individuals (Hedge's *g* = −0.427) (38), which indicates that 2D:4D is more strongly related to addiction than to the frequency or amount of use. Moreover, lower 2D:4D associates with greater liver, muscle, and myelotoxic effects of alcohol and prospective hospital readmission in dependent patients (22). Alcohol-dependent males with lower 2D:4D are also more willing to purchase higher-priced alcoholic drinks (23). In parallel, alcohol-dependent patients (22) and individuals reporting binge drinking behavior (39) also report later age at spermarche. Experimental animal data show that prenatal androgen treatment increases pubertal onset age in male rats (35). Taken together, these data indicate that higher prenatal androgen exposure predisposes an individual to develop and maintain addictive disorders during adulthood. Interestingly, recent work suggests that stress, smoking, and alcohol use during pregnancy increases prenatal testosterone exposure, as indicated by lower 2D:4D in the human offspring (22, 40). Thus, maternal behavior might be an effective, novel target for addiction prevention among her offspring (41).

Alcohol use disorder and the problematic use of pornography overlap greatly in several aspects, which suggests common etiopathogenetic mechanisms (42). Sex-related rewards not only converge on the same neural pathway as drug rewards, but they also share the same molecular mediators and, most likely, the same neurons in the NAc, in contrast to other natural rewards like food (43). The incentive-salience model of addiction fits well with the dissociation observed in pornography addiction of increased craving (“wanting”) and decreased pleasure from use (“liking”) (44). Interestingly, especially the expectation to feel high following alcohol consumption correlates with lower 2D:4D (23). In addition to the molecular predispositions to addiction, pornography use might be more attractive for men with lower 2D:4D, as they have higher isolation intolerance (45), show more aggression or dominance behavior in some situations (46), and are more status-oriented (47). However, the role of intrauterine androgen level in online sexual compulsivity (OSC) and its related sexual dysfunctions have not yet been studied. Therefore, we tested our primary hypotheses that lower 2D:4D and later age at spermarche are related to OSC.

In addition to the reward system-related influences of prenatal androgen levels, prenatal androgen exposure shapes reproductive organs; i.e., lower 2D:4D (higher prenatal testosterone) correlates with greater penile length (48) and larger testes (49). Lower prenatal testosterone feminizes the reproductive organs (50, 51). Moreover, individuals with lifelong premature ejaculation have lower 2D:4D (52). Therefore, we also investigated whether 2D:4D and age at spermarche are associated with erectile function and/or ejaculatory control.

## Methods

### Demographic Data

The data analyzed here originated from the first to third survey waves of the longitudinal Cohort Study on Substance Use Risk Factors (C-SURF; www.c-surf.ch). From 2010 to 2012, 7,556 young males attending mandatory recruitment for the Swiss army provided written informed consent, of whom 5,987 men participated in Wave 1. In Wave 2, 5,036 males completed the questionnaire from 2012 to 2013, and Wave 3 spanned from 2016 to 2018 and included 5,160 males (see www.c-surf.ch). All analyzed data originated from Wave 3, except for the ejaculatory control and erectile function variables, which were assessed in Waves 1 and 2 only. We included young males who reported only being attracted to women, for several reasons: first, we wanted to maximize the homogeneity of our sample in terms of sexual behavior; second, one item was formulated specifically for vaginal penetration in the German version.

### 2D:4D

Similar to the methods described by (53) and (39), the participants were instructed to self-measure their 2D:4D (Questionnaire No. 3 ID: J18). They documented the lengths of the index and ring fingers in millimeters for their right and left hands separately. To eliminate inaccurate values, finger lengths below 10 mm and above 100 mm (53) and, subsequently, 2D:4D outside of the 2.5 and 97.5 percentiles (39, 54) were excluded, as previously described. We selected the mean of the right-hand and left-hand 2D:4D (Mean2D:4D) as the primary predictor and right-hand 2D:4D (R2D:4D), left-hand 2D:4D (L2D:4D), and the difference between R2D:4D and L2D:4D (2D:4Dr-l) as exploratory predictors.

### Pubertal Onset Age

Self-reported pubertal onset age was controlled for time passed (years passed by since puberty) using partial correlation analysis, as recall biases are prevalent (55), i.e., the variance in the variable age at puberty onset that correlated with years since puberty (current age-puberty age) was removed. Furthermore, estimates below 9 were excluded, based on a previous report (56) and a previous analysis of 2D:4D and pubertal onset age (22).

### OSC

The Internet Sex Screening Test (ISST; http://www.recoveryzone.com/tests/sex-addiction/ISST/index.php, developed by Delmonico, 1997) is a self-administered screening instrument that identifies clinically problematic sexual internet-based behavior. Factor analysis of the ISST data identified five factors: OSC, online sexual behavior-social, online sexual behavior-isolated, online sexual spending, and interest in online sexual behavior (57). The OSC subscale was included in the C-SURF questionnaire, consisting of six binary (yes/no) item. Subjects who did not visit a pornographic web site within the past 12 months (22.4%, *n* = 1,064) were excluded from the analysis. As clinically relevant cut-off scores do not yet exist and little research is available on the matter, we decided to use the sum score as a continuous variable in our analysis.

### Pornography Consumption

Data from two items were available: one on the frequency of use (i.e., consumption days per month) and one on the duration of each use. In our cohort, the interquartile range (IQR) of consumption days was 3 to 15 days per month. Duration of the use: almost none, 1 to <2 h, 2 to <3 h, 3 to <4 h, 4 h, or more. We considered frequency to be more informative here, as the variability in consumption time was low, with 90% self-reporting <1 h.

### Erectile Function

The International Index of Erectile Function (IIEF-5) Questionnaire consists of five items, scored using a five-point Likert scale. How do you rate your confidence that you could get and keep an erection? When you had erections with sexual stimulation, how often were your erections hard enough for penetration (entering of the penis into the vagina)? During sexual intercourse, how often were you able to maintain your erection after you had penetrated your partner? During sexual intercourse, how difficult was it to maintain your erection to completion of intercourse? When you attempted sexual intercourse, how often was it satisfactory for you? The sum score was coded as a continuous variable for correlation analysis.

### Ejaculatory Control

One item (five-point Likert scale) from the Premature Ejaculation Prevalence and Attitude (PEPA) survey was used (58): Within the last 6 months, how do you rate your control over ejaculation during partnered sex?

### Ethical Approval

All subjects provided written informed consent prior to their inclusion in the original study. This study was approved by the Ethics Committee for Clinical Research of Lausanne University Medical School (Protocol No. 15/07).

### Statistical Analyses

All data were analyzed using IBM SPSS Statistics version 24 for Windows (SPSS Inc., Chicago, IL, USA). When data points were missing, the study subject was excluded from the specific analysis (the number of individuals included in each analysis is reported as *N*). Descriptive statistics were expressed in frequencies, medians, and IQRs. We used the Wilcoxon signed-rank test to compare the dependent groups. Correlations were identified using Spearman's rank method, as the data were not normally distributed. *p* < 0.05 was considered to be statistically significant for two-sided tests. Semipartial correlations between residuals were performed to reveal the specific links connecting the variables. As described below, we also dissociated consumption-frequency-related effects from reported compulsivity by semi-partial correlations as a *post-hoc* analysis.

## Results

### Cohort Demographics

After the step-wise exclusion of subjects who failed to meet the quality criteria of 2D:4D (*n* = 518) and/or pubertal onset age (*N* = 94) and who were not exclusively attracted to women (*N* = 534), the total cohort was characterized as follows: age 25 years (IQR 25–26, *N* = 4,370); body mass index 23.6 kg/m^2^ (IQR 21.9–25.5, *N* = 4,362); 79.8% gainfully employed (*N* = 4,369); education: 3.0% secondary education, 1.2% basic vocational education, 34.9% secondary vocational/technical education, 4.4% community college, 11.1% vocational high school, 11.3% high school, 23.2% bachelor degree (university), 5.9% masters degree (university), 4.7% other (*N* = 4,358); marital status: 82.9% single, 5.3% married, 0.1% divorced, 11.5% not married, separated, or divorced but living together with a partner (e.g., in a registered partnership), 0.2% married but separated, 0.0% widowed (*N* = 4,363); 37.5% were still living with their parent(s). In the last 12 months, 59.9% had one sexual partner, 5.9% had none, 34.2% had two or more. Mean2D:4D was 0.981 (IQR 0.955–1.000, *N* = 4,177), R2D:4D 0.986 (IQR 0.951–1.000, *N* = 4,269), L2D:4D 0.986 (IQR 0.951–1.000 *N* = 4,278), 2D:4Dr-l 0.000 (IQR −0.013–0.012, *N* = 4,177).

Of the pornography-consuming subjects, 41% gave at least one positive response to the OSC questions; 18.4% reported at least two problematic behaviors from the OSC. In our cohort, 41.3% reported at least mild erection problems, and 5% reported poor control over ejaculation during intercourse.

### Prenatal Testosterone Markers and OSC

First, we tested our main hypothesis, stating that increased prenatal testosterone, as indicated by a lower Mean2D:4D and/or higher pubertal onset age, is associated with a higher OSC score in our cohort. While Mean2D:4D correlated significantly in the expected direction, the self-reported pubertal onset age did not (Table 1).

**Table 1:** Correlation between prenatal testosterone markers and OSC.

			**OSC**	**OSC (controlled)**
Spearman-Rho	Mean2D:4D	Coefficient	−0.042	−0.044
		*p*	**0.015**	**0.011**
		*N*	3276	3273
	Pubertal onset age	Coefficient	−0.010	0.048
		*p*	0.556	**0.004**
		*N*	3682	3678

*2D:4D, second-to-fourth-finger length ratio; Mean2D:4D, mean of right-hand and left-hand 2D:4D; OSC, online sexual compulsivity; OSC (controlled), variance that correlated with consumption removed; p < 0.05 in bold print*.

Next, we controlled for the actual consumption frequency in our dependent variable OSC, as more severe compulsivity was associated with increased use (Rho = 0.184, *p* < 0.001, *N* = 3,678), pubertal onset age was negatively correlated with consumption frequency (Rho = −0.124, *p* < 0.001, *N* = 3,680), but Mean2D:4D was not (Rho = 0.008, *p* = 0.647, *N* = 3,274) and we were specifically interested in the compulsivity aspect, given a certain consumption level. After correcting for the frequency of use, the OSC score correlated negatively with Mean2D:4D and positively with pubertal onset age (both indicative of higher prenatal testosterone level), thus supporting our primary hypothesis (Table 1).

In a *post-hoc* analysis, we explored relationships of OSC scores with R2D:4D, L2D:4D, and 2D:4Dr-l (Table 2). L2D:4D correlated significantly with OSC, while only a trend was observed for R2D:4D.

**Table 2:** *Post hoc* analysis of 2D:4D markers.

			**OSC**	**OSC (controlled)**
Spearman-Rho	L2D:4D	Coefficient	−0.037	−0.037
		*p*	**0.033**	**0.031**
		*N*	3348	3345
	R2D:4D	Coefficient	−0032	−0.032
		*p*	0.064	0.066
		*N*	3348	3345
	2D:4Dr-l	Coefficient	0.019	0.014
		*p*	0.285	0.415
		*N*	3276	3273

*2D:4D, second-to-fourth-finger length ratio; exploratory predictors: L2D:4D, left-hand 2D:4D; R2D:4D, right-hand 2D:4D; 2D:4Dr-l, difference between R2D:4D and L2D:4D; OSC, online sexual compulsivity; OSC (controlled), variance that correlated with consumption removed; p < 0.05 in bold print*.

As vulnerability for mood disorders and traits like sensation seeking might be influenced by prenatal as well as pubertal androgen exposure which could mediate some of the observed effects, we performed an exploratory analysis on the available scores for major depression, MDI (59), bipolar disorder, MDQ (60), and sensation seeking, BSSS (61). Whereas Mean2D:4D did not significantly correlate with these measures respectively (Rho = −0.002, *p* = 0.922, *N* = 4,155; Rho = −0.015, *p* = 0.335, *N* = 4,161; Rho = 0.006, *p* = 0.698, *N* = 4,170), higher puberty onset age was associated with a lower number of symptoms respectively (Rho = −0.032, *p* = 0.029, *N* = 4,717; Rho = −0.050, *p* = 0.001, *N* = 4,720) and less sensation seeking (Rho = −0.118, *p* < 0.001, *N* = 4,736).

### Prenatal Testosterone Markers and Sexual Dysfunction

To investigate the influence of prenatal testosterone on sexual dysfunction and test our secondary hypotheses, we first explored the development of ejaculatory control and erectile function over time (i.e., from Wave 1 to Wave 2, since sexual dysfunction was not assessed in Wave 3). There was a significant increase in erectile function over time but no change in ejaculatory control (*Z* = −5.76, *p* < 0.001; *Z* = −2.15, *p* = 0.830). Therefore, we controlled our dependent variable erectile function (from Wave 2) for age. Pubertal onset age correlated negatively with erectile function (controlled) but not with ejaculatory control; Mean2D:4D did not correlate significantly with either; see Table 3.

**Table 3:** Prenatal testosterone markers and sexual functions.

			**Erectile function (controlled)**	**Ejaculatory control**
Spearman-Rho	Mean2D:4D	Coefficient	−0.012	0.002
		*p*	0.501	0.904
		*N*	3321	3325
	Pubertal onset age	Coefficient	−0.143	−0.005
		*p*	**<0.001**	0.782
		*N*	3771	3781

*2D:4D, second-to-fourth-finger length ratio; Mean2D:4D; controlled, variance that correlated with age removed; p < 0.05 in bold print*.

Given suggestions in the literature that pornography consumption influences sexual dysfunction, we explored the relationships between pornography use, OSC, and sexual functions. Interestingly, pornography use frequency did not significantly correlate with erectile function, whereas OSC did, with more compulsive symptoms related to less ejaculatory control and less erectile function (Table 4); moreover, the hours spent on pornography in each occasion did not correlate significantly with either.

**Table 4:** Pornography use and sexual functions.

			**Erectile function (controlled)**	**Ejaculatory control**
Spearman-Rho	OSC	Coefficient	−0.128	−0.062
		*p*	**<0.001**	**0.002**
		*N*	2949	2599
	OSC (controlled)	Coefficient	−0.129	−0.062
		*p*	**<0.001**	**0.002**
		*N*	2945	2596
	Frequency of use (days/month)	Coefficient	0.011	−0.004
		*p*	0.540	0.829
		*N*	2946	2596
	Hours of use (on days of use)	Coefficient	−0.014	−0.016
		*p*	0.444	0.414
		*N*	2949	2598

*Erectile function (controlled), variance that correlated with age removed; OSC, online sexual compulsivity; OSC (controlled), variance that correlated with consumption removed; p < 0.05 in bold print*.

## Discussion

Here we describe the first evidence of the influence of prenatal androgen exposure on OSC behavior in males during young adulthood. Our data confirmed our primary hypotheses that lower 2D:4D and later age at spermarche—both independently indicative of higher prenatal testosterone levels—were significantly (although with small effect size) associated with stronger OSC, despite reliable measurements of finger length from multiple expert raters and clinical data on time of puberty onset being unavailable.

These findings align well with the existing knowledge. The male sexual response and associated natural reward are mediated *via* mesolimbic dopamine signaling in the VTA and the NAc (8). This circuit forms the core of the reward system and, as such, it does not only mediate sexual reward (62) but also underlies substance addictions, such as alcoholism (63). Prenatal testosterone is suggested to affect the onset and course of alcohol dependence (22), and a study in mice found that prenatal modulation of androgen receptors affects cerebral dopamine, serotonin, and noradrenaline neurotransmitter levels in adulthood (29). In female sheep, prenatal testosterone positively correlates with the number of tyrosine hydroxylase-immunoreactive cells in the VTA (64). Furthermore, methamphetamine addiction is also mediated by the same neural substrates as sexual stimulation (65). Repeated sexual behaviors and repeated psychostimulant administration both induce the up-regulation of DeltaFosB, thereby sensitizing the mesolimbic pathway (43). Gene expression of the mu-opioid receptor, a key player in addiction pathology, appears to be sex-specifically altered by prenatal testosterone intervention (29). Moreover, the A118G variant of the mu-opioid receptor gene interacts with 2D:4D to predict alcohol dependence (66).

Whereas, OSC was associated with higher prenatal testosterone levels indicated by both markers, use frequency showed the opposite relationship with pubertal onset age, which might be a social peer group effect. A recent meta-analysis also concluded that 2D:4D relates more to addiction phenotypes than the frequency or amount of use (38). In summary, our findings both reinforce and further our understanding of drug addiction and addiction to sexual reward, namely, that they may share the same neural circuits that are vulnerable to prenatal androgen levels.

Our secondary hypothesis, that increased prenatal testosterone may also affect sexual functions, was only partially supported by the data. We found a significant correlation between erectile function and time of puberty, with later onset being associated with less function; however, we did not find a link to Mean2D:4D. This inconsistency may be due to the different prenatal windows during which 2D:4D and pubertal timing are determined. Two independent studies have provided evidence of 2D:4D development occurring during early pregnancy (67, 68). In contrast, when pubertal timing is exactly determined remains unclear, and it can be assumed that pubertal timing is not only a marker for prenatal androgen exposure but also influences brain organization during adolescence.

Additional research is needed to clarify whether the organizational influence of prenatal androgen on the reward system mediates this link, whether increased peripheral androgen receptors, which are involved in erectile function (69) play a role, or whether erectile dysfunction is a secondary effect of OSC and, therefore, arises from increased consumption of pornographic content and impacts sexual arousal during partnered sex *via* associated motivational aspects.

In the future, validated screening tools are required to disentangle the origins of sexual dysfunction related to pornography addiction by accurately assessing the context of sexual difficulties, progression of OSC, and pornography consumption over time. Also, developmental factors should be considered, as the reward circuit and its prefrontal control are highly vulnerable during adolescence (70). Additionally, experimental manipulation of consumption frequency, clinical interventions based on pornography abstinence, and investigation of pharmacological effects on dysfunction should be carefully investigated in the future, to further the understanding of the underlying etiology.

Ejaculatory control did not correlate with either prenatal testosterone marker. Given a previous study reporting a link between prenatal testosterone and premature ejaculation (52), this finding was initially unexpected. However, the cohort involved in that study differed from ours in several ways. First, the Bolat et al. (52) study only included patients with a lifelong history of premature ejaculation issues. Second, their cohort was older (mean age 40 years). Third, we do not know how experienced the subjects of our study were in controlling ejaculation during intercourse, as 82% are single, which limits experiential learning with a confidant. Fourth, pornography-related behavior was not assessed in our study.

Pornography-related sexual dysfunctions are not yet well-understood. A recent review describes pornography, its availability, and many different forms as a supernatural stimulus, which, in the long-term, leads to problems achieving sufficient stimulation in natural (partnered) settings. This, in turn, can cause several issues, from erectile dysfunction during partnered intercourse and delayed ejaculation, to being unable to ejaculate entirely during partnered sex (3). We did not have sufficient data in the present study to distinguish between premature and delayed ejaculation, as both are covered by the item about ejaculatory control, which was negatively associated with OSC. A recently published model describing users' need for more extreme material over time to be able to ejaculate has not yet been verified (71), and increased tolerance is currently not yet well-defined for pornography addiction. However, pornography consumption influences subjective and self-reported estimates of typical latency times.

We find it very interesting that OSC, not pornography use itself, was associated with less ejaculatory control and less erectile function; this suggests a tight link between OSC and sexual dysfunction *via* alterations to the reward system as opposed to social associative mechanisms. Also here, more research is needed to disentangle cause and effect.

The present study is subject to several limitations. 2D:4D was self-quantified, and frequencies of pornography use, erectile function, and ejaculatory control were self-reported. Pornography addiction is not yet formally recognized as a behavioral addiction, and, therefore, its definition varies (72). Here, we focused on the OSC subscale of the ISST, representing the compulsivity aspect of this behavioral addiction. Moreover, we investigated a homogeneous cohort of young, heterosexual males, most of whom were Caucasian and single; therefore, our findings cannot be generalized to other age groups, sexual orientations, ethnicities, or females. Finally, 2D:4D and puberty onset have limited validity as markers for prenatal androgen exposure (33, 38, 73), and it is likely that pubertal timing also directly affects brain organization, as puberty is also a sensitive time window (74). Therefore, our finding of an association between pubertal timing and OSC may not only be a result of prenatal but also pubertal androgen exposure associated vulnerabilities.

In conclusion, higher prenatal androgen levels (indicated by two independent markers) are associated with more compulsive pornography use. A more compulsive use in turn is associated with less erectile function and low ejaculatory control in young men. In addition, less erectile function was associated with a higher pubertal onset age, which may indicate higher prenatal androgen levels. Thus, the etiology of erectile dysfunction and its sharp rise in prevalence within the last decade might involve an interaction of a prenatal predisposition to develop sexual online compulsivity and/or erectile dysfunction and increased availability of pornographic content. Future studies are encouraged to disentangle the relative contribution of these factors and further the understanding of this behavioral addiction and related sexual problems. These insights could help to develop prevention programs, targeting either subjects at risk to develop this addiction or mothers whose prenatal testosterone levels are high.

## Data Availability Statement

The datasets generated for this study are available on request to the corresponding author.

## Ethics Statement

The studies involving human participants were reviewed and approved by Ethics Committee for Clinical Research of Lausanne University Medical School (Protocol No. 15/07). The patients/participants provided their written informed consent to participate in this study.

### Members of the Cohort Study on Substance Use Risk Factors

Gerhard Gmel: Addiction Medicine, Lausanne University Hospital CHUV, University of Lausanne, Lausanne, Switzerland; Addiction Switzerland, Lausanne, Switzerland; Centre for Addiction and Mental Health, Toronto, ON, Canada; University of the West of England, Frenchay Campus, Bristol, United Kingdom (Gerhard.Gmel@chuv.ch). Meichun Mohler-Kuo: La Source, School of Nursing Sciences, HES-SO University of Applied Sciences and Arts of Western Switzerland, Lausanne, Switzerland (m.mohler-kuo@ecolelasource.ch). Simon Foster: Institut für Epidemiologie, Biostatistik und Prävention, Hirschengraben, Zürich, Switzerland (simon.foster@kjpd.uzh.ch). Simon Marmet: Addiction Medicine, Lausanne University Hospital CHUV, University of Lausanne, Lausanne, Switzerland (simon.marmet@chuv.ch). Joseph Studer: Addiction Medicine, Lausanne University Hospital CHUV, University of Lausanne, Lausanne, Switzerland (Joseph.Studer@chuv.ch).

## Author Contributions

VB and BL conceived and designed the research, analyzed the data, and wrote the manuscript. GG, MM, SM, SF, and JS performed the experiments. CM and JK commented on the manuscript and provided the intellectual input. All authors contributed to the article and approved the submitted version.

## Conflict of Interest

The authors declare that the research was conducted in the absence of any commercial or financial relationships that could be construed as a potential conflict of interest. The handling editor declared a shared affiliation with one of the authors GG at time of review.
